# Proteomic analysis of shoot tissue during photoperiod induced growth cessation in *V. riparia *Michx. grapevines

**DOI:** 10.1186/1477-5956-8-44

**Published:** 2010-08-12

**Authors:** Kim J Victor, Anne Y Fennell, Jérôme Grimplet

**Affiliations:** 1 Department of Horticulture, Forestry, Landscape, & Parks, Box 2140A, South Dakota State University, Brookings, SD 57007, USA; 2 Instituto de Ciencias de la Vid y del Vino (CSIC, Universidad de La Rioja, Gobierno de La Rioja) Longroño, 26006, Spain

## Abstract

**Background:**

Growth cessation, cold acclimation and dormancy induction in grapevines and other woody perennial plants native to temperate continental climates is frequently triggered by short photoperiods. The early induction of these processes by photoperiod promotes winter survival of grapevines in cold temperate zones. Examining the molecular processes, in particular the proteomic changes in the shoot, will provide greater insight into the signaling cascade that initiates growth cessation and dormancy induction. To begin understanding transduction of the photoperiod signal, *Vitis riparia *Michx. grapevines that had grown for 35 days in long photoperiod (long day, LD, 15 h) were subjected to either a continued LD or a short photoperiod (short day, SD, 13 h) treatment. Shoot tips (4-node shoot terminals) were collected from each treatment at 7 and 28 days of LD and SD for proteomic analysis via two-dimensional (2D) gel electrophoresis.

**Results:**

Protein profiles were characterized in *V. riparia *shoot tips during active growth or SD induced growth cessation to examine physiological alterations in response to differential photoperiod treatments. A total of 1054 protein spots were present on the 2D gels. Among the 1054 proteins, 216 showed differential abundance between LD and SD (≥ two-fold ratio, p-value ≤ 0.05). After 7 days, 39 protein spots were more abundant in LD and 30 were more abundant in SD. After 28 days, 93 protein spots were more abundant in LD and 54 were more abundant in SD. MS/MS spectrometry was performed to determine the functions of the differentially abundant proteins.

**Conclusions:**

The proteomics analysis uncovered a portion of the signal transduction involved in *V. riparia *grapevine growth cessation and dormancy induction. Different enzymes of the Calvin-Benson cycle and glutamate synthetase isoforms were more abundant either in LD or SD treatments. In LD tissues the significantly differentially more abundant proteins included flavonoid biosynthesis and polyphenol enzymes, cinnamyl alcohol dehydrogenase, and TCP-1 complexes. In the SD tissue photorespiratory proteins were more abundant than in the LD. The significantly differentially more abundant proteins in SD were involved in ascorbate biosynthesis, photosystem II and photosystem I subunits, light harvesting complexes, and carboxylation enzymes.

## Background

Viticulture and enology have a rich history beginning over 7,000 years ago. With the growth of civilization grapevines became a prominent fruit crop and are now the most widely grown and economically important in the world. Even though the majority of wine production takes place in Mediterranean or oceanic climate areas, vineyards of continental regions contribute greatly to the diversity of viticulture. Grapevines grown in these temperate climates must be adapted to cold, dry winters in order to survive. *Vitis riparia*, the only grape species native to the upper Midwest region of the United States, is particularly adapted to colder climates [1,2].

Like many perennial plants, grapevines survive subzero winter temperatures by ceasing growth and entering dormancy. In many temperate woody species, the transition from active growth to dormancy is promoted by decreasing daylength [3]. Photoperiodic response is a stable annual cue that provides plants with a reliable timing mechanism to signal winter's onset [4]. Daylength sensing takes place in the leaves and a signal is believed to be transported to the shoot apex [5]. In tree species with photoperiodically induced dormancy, such as birch (*Betula*), the perception of decreasing daylengths results in cessation of growth, development of a terminal bud, and progression to a dormant and more freezing-tolerant state [6,7]. The decreased daylength also triggers other adaptive responses including nitrogen storage, stem growth cessation, and leaf senescence [8].

In contrast to tree species such as poplar (*Populus*) and birch, *V. riparia *does not set a terminal bud in response to decreasing daylength in the autumn. Upon reaching a critical daylength specific to a given *V. riparia *ecotype, shoot growth ceases and shoot tip abscission and latent bud dormancy are induced [9-11]. Shoot tip abscission coincides with bud dormancy induction in grapevines and occurs prior to leaf senescence. Full shoot tip abscission in *V. riparia *takes place after 28 days of short photoperiod (SD) [11]. The shoot tip begins to yellow from the 2^nd ^node to the apex and eventually dries up and falls from the plant. Autumn senescence, or programmed cell death, stimulates many changes in gene expression which are accompanied by a remobilization of nutrients, carbohydrate accumulation, and shedding of plant parts [12]. This study examined protein abundance during the transition from active growth to initiation of shoot tip abscission to begin unraveling SD programmed induction of growth cessation and shoot tip senescence in grapevines. Quantitative and qualitative differences in protein abundance were identified by employing a phenol-based extraction and 2D gel analysis [13,14].

## Results

### Photoperiod regulation of shoot growth

**Figure 1:**
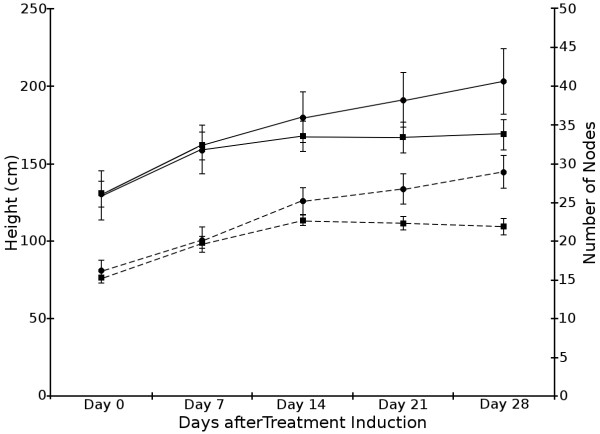
**LD vs. SD physiological data**. Primary shoot length and node number were determined for LD (circle) and SD (square) treatments at various time points. Solid lines indicate primary shoot length; dashed lines represent node number.

Measurements of *V. riparia *primary shoot length and node number were initiated at day zero of the differential photoperiod treatments and were repeated every seven days (0, 7, 14, 21, and 28 days). Figure 1 shows that shoot length and node number were similar for the first 7 days of LD and SD. At day 14, the shoot length and node number were statistically different (p-value ≤ 0.05 and ≤ 0.001 respectively) between LD and SD treatments. By 28 days growth had ceased in the SD vines and there was a decrease in node number as tip abscission occurred. The LD treated *V. riparia *grapevines continued to grow and had a greater total shoot length and node number.

### Differential photoperiod influence on protein abundance

2D PAGE analysis was used to examine the response to photoperiod change and the physiological alterations as the shoot tip yellows and abscission is initiated. Proteins were extracted from four node shoots harvested after 7 and 28 days of differential photoperiod treatment (six replicates for each time point and photoperiod). There was no significant difference in the amount of recovered proteins that was observed between photoperiod treatments from the same harvest time point. Total protein recovery averaged 5.4 ± 1.4 mg per gram of tissue extracted.

In total, 1054 spots were detected among the four treatments sampled (two photoperiods × two time points). An average of 785 spots per gel with an intensity value greater than 0.01% of the total average spot intensity was observed. Faint spots were included in the gel analysis to maximize the number of proteins identified and to increase the potential of indentifying signaling-related proteins that are typically low in abundance. The inclusion of faint spots increased spot number per gel but also resulted in a comparatively high average coefficient of variation (CV) (7SD: 0.76; 7LD: 0.82; 28SD: 0.70; 28LD: 0.72). However, these CV values were within a range consistent with values previously reported for other plant proteomic analyses (0.26-0.31)[15] (0.47-0.75) [16] and (0.24) [17].

No significant difference in the number of spots with an intensity greater than 0.01% was observed at 7 days of differential photoperiod treatment. At 28 days, the LD treatment presented a significantly (p value = 0.0002) greater number of spots than the SD treatment (814 versus 742 spots). A few major proteins may have contributed to these differences as in 28LD where the top 10 most intense proteins accounted for 10.2% of the total spot intensity while in 28SD the top 10 most intense proteins accounted for 13.7% of the total spot intensity.

**Figure 2:**
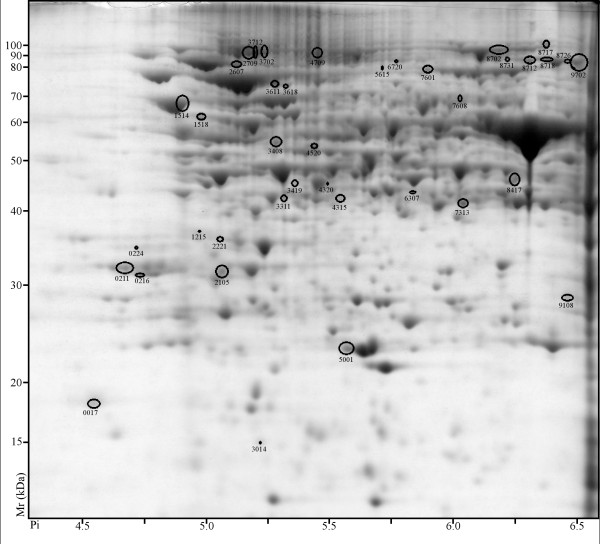
**2D PAGE analysis of *V. riparia *after 7 days of LD treatment**. Proteins that exhibited a significant change (≥ two-fold ratio, p-value ≤ 0.05) between LD and SD are indicated by circles and standard spot numbers on a representative replicate gel. See Table 1 for a detailed listing of proteins.

**Figure 3:**
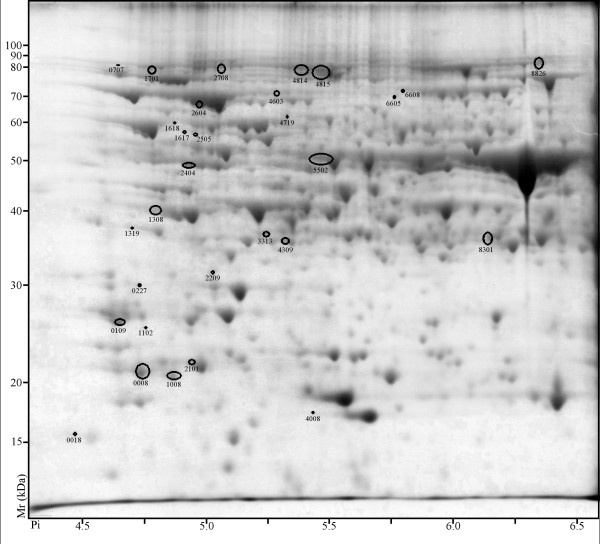
**2D PAGE analysis of *V.riparia *after 7 days of SD treatment**. Proteins that exhibited a significant change (≥ two-fold ratio, p-value ≤ 0.05) between LD and SD are indicated by circles and standard spot numbers on a representative replicate gel. See Table 2 for a detailed listing of proteins.

**Figure 4:**
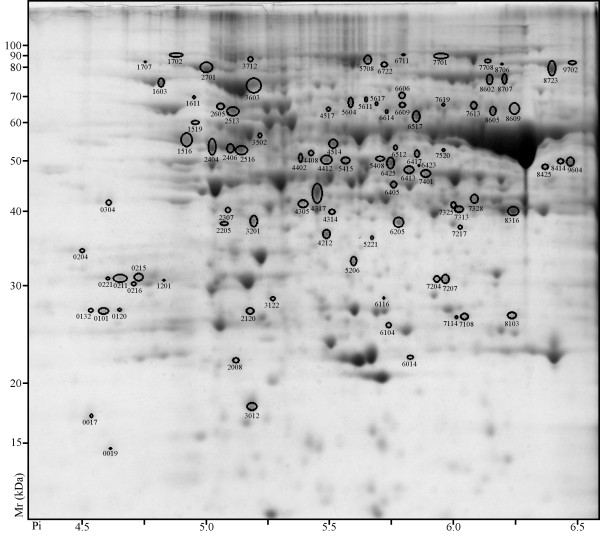
**2D PAGE analysis of *V. riparia *after 28 days of LD treatment**. Proteins that exhibited a significant change (≥ two-fold ratio, p-value ≤ 0.05) between LD and SD are indicated by circles and standard spot numbers on a representative replicate gel. See Table 3 for a detailed listing of proteins.

**Figure 5:**
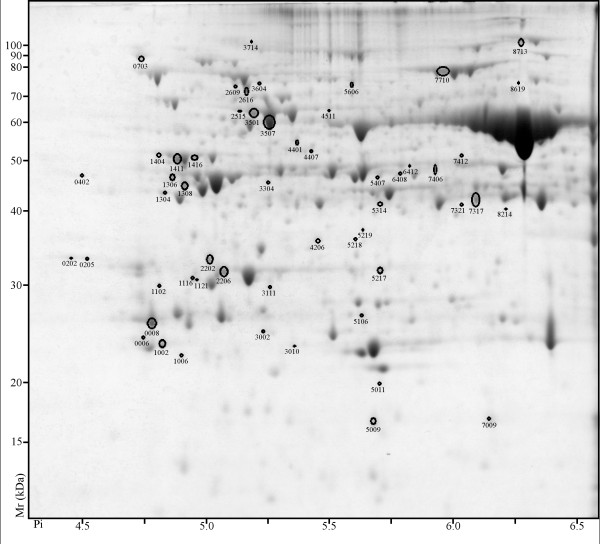
**2D PAGE analysis of *V. riparia *after 28 days of SD treatment**. Proteins that exhibited a significant change (≥ two-fold ratio, p-value ≤ 0.05) between LD and SD are indicated by circles and standard spot numbers on a representative replicate gel. See Table 4 for a detailed listing of proteins.

Proteome differences were analyzed at 7 days and 28 days of differential photoperiod treatment. At 7 days 69 spots displayed differential abundance (ANOVA, p-value ≤0.05) and ≥ two-fold ratio. Of these 69 spots, 39 were more abundant in LD (Figure 2) and 30 were more abundant in SD (Figure 3). At 28 days, 147 spots displayed differential abundance (ANOVA, p-value ≤0.05) and ≥ two-fold ratio. Of these 147 spots, 93 were more abundant in LD (Figure 4) and 54 were more abundant in SD (Figure 5).

### Identification of differentially abundant proteins between photoperiod treatments

Protein spots that displayed differential abundance (ANOVA, p-value ≤0.05) and ≥ two-fold ratio between the two photoperiods were excised and analyzed by MALDI TOF/TOF. At 7 days, 68 of the 69 differentially abundant protein spots were positively identified (Table 1 and 2). At 28 days, 137 of the 147 differentially abundant protein spots were positively identified (Table 3 and 4). The identity of the majority of the protein spots was determined using the putative proteins from the homozygote Pinot Noir (PN40024) genome sequence; only 4 were identified from different *Vitis *data sources (tentative contig, EST or the heterozygote genome). Supplementary spot data are available in Additional File 1; including IDs of corresponding predicted proteins from genome sequencing data, data for other proteins with positive IDs, and abundance of each spot on each replicate gel.

**Table 1:** Proteins whose abundance was significantly more in LD than SD at 7 days.

SSP	SD/LD	Pval	Th Mr	Exp Mr	Th Pi	Exp Pi	Pep	M score	% Cov	Function
SSP8731	0.34	0.00	85	83	6.1	6.1	22 (11)	592	35	5-Me-tetrahydropteroyltriglu-homocys S-Me-transferase
SSP8726	0.09	0.03	85	83	6.1	6.3	29 (12)	829	43	5-Me-tetrahydropteroyltriglu-homocys S-Me-transferase
SSP9702	0.10	0.03	85	83	6.1	6.4	25 (13)	783	39	5-Me-tetrahydropteroyltriglu-homocys S-Me-transferase
SSP8718	0.14	0.01	85	83	6.1	6.2	29 (13)	845	44	5-Me-tetrahydropteroyltriglu-homocys S-Me-transferase
SSP4320	0.13	0.02	37	44	5.4	5.5	12 (8)	365	46	Aspartate-semialdehyde dehydrogenase*
SSP8717	0.42	0.01	115	94	6.9	6.2	29 (15)	819	35	Glycine dehydrogenase
SSP6307	0.24	0.03	32	43	6.2	5.8	12 (2)	95	45	Cysteine synthase
SSP3311	0.18	0.04	39	42	5.4	5.3	14 (8)	572	54	Glutamine synthetase*
SSP4315	0.11	0.01	39	42	5.7	5.5	7 (5)	293	21	Glutamine synthetase cytosolic*
SSP7608	0.09	0.00	65	65	5.9	6.0	17 (9)	462	35	Pyruvate decarboxylase isozyme 2
SSP9108	0.08	0.05	27	28	6.3	6.3	11 (5)	416	64	Triosephosphate isomerase, cytosolic
SSP7601	0.43	0.01	67	77	6.0	5.8	20 (9)	316	47	Transketolase, chloroplast precursor
SSP1518	0.42	0.01	59	60	6.6	5.0	24 (11)	626	51	ATP synthase beta chain 2, mitochondrial
SSP2105	0.15	0.00	32	30	6.0	5.1	11 (8)	331	42	Inorganic pyrophosphatase*
SSP3611	0.07	0.04	69	72	5.2	5.3	29 (12)	448	52	V-type H+-transporting ATPase subunit A
SSP3618	0.13	0.02	69	71	5.2	5.3	35 (13)	668	60	V-type H+-transporting ATPase subunit A
SSP4709	0.18	0.03	102	88	6.1	5.4	22 (8)	290	25	Lipoxygenase (LOX2)*
SSP7313	0.46	0.01	37	41	5.8	5.9	12 (10)	517	28	Anthocyanidin reductase
SSP8417	0.29	0.01	43	46	6.1	6.1	19 (10)	515	47	Chalcone synthase (CHS)
SSP2221	0.13	0.01	31	35	5.0	5.1	11 (10)	468	43	Proteasome 20 S alpha subunit F2*
SSP4520	0.16	0.04	47	52	5.4	5.4	25 (14)	887	63	Proteasome 26 S AAA-ATPase subunit RPT3*
SSP1514	0.41	0.02	62	64	5.1	4.9	23 (18)	1330	53	60 kDa chaperonin alpha subunit
SSP0224	0.03	0.03	30	33	4.7	4.7	28 (10)	589	63	14-3-3 protein GF14 nu (GRF7)
SSP0216	0.09	0.01	29	30	4.8	4.7	23 (10)	554	51	14-3-3 protein GF14 omega (GRF2)
SSP0211	0.30	0.00	29	31	4.7	4.7	24 (10)	562	59	14-3-3 protein GF14 omega (GRF2)
SSP2709	0.26	0.00	90	86	5.1	5.1	38 (17)	1110	49	Cell division cycle protein 48
SSP3702	0.09	0.01	90	86	5.1	5.2	36 (17)	1060	47	Cell division cycle protein 48
SSP3712	0.16	0.00	90	86	5.1	5.2	36 (17)	1080	47	Cell division cycle protein 48
SSP5001	0.27	0.01	17	22	5.7	5.5	6 (3)	419	42	Abscisic stress ripening protein 2 (ASR2)
SSP6720	0.18	0.04	65	82	5.0	5.7	22 (6)	283	37	Heat shock protein 81-4
SSP8702	0.06	0.03	56	90	6.1	6.1	15 (8)	330	34	Elongation factor EF-2*
SSP2607	0.24	0.02	75	79	5.0	5.1	32 (14)	843	50	Elongation factor G, chloroplast precursor
SSP3408	0.29	0.02	47	53	5.4	5.3	33 (12)	534	61	Eukaryotic initiation factor 4A
SSP8712	0.18	0.00	83	83	6.4	6.2	29 (8)	443	45	EMB1138 (Embryo defective 1138)*
SSP3014	0.08	0.03	16	14	6.3	5.2	8 (7)	269	63	GRP7 (cold, circadian rhythm, and RNA binding 2) *
SSP5615	0.12	0.03	84	78	5.8	5.6	25 (4)	269	35	Protein transport protein Sec23A
SSP1215	0.06	0.01	40	36	5.0	5.0	15 (8)	430	60	Lipase GDSL
SSP0017	0.03	0.00	19	17	4.5	4.6	4 (3)	117	20	Translationally-controlled tumor protein
SSP3419	0.18	0.03	40	44	5.8	5.3	16 (8)	350	37	Unknown protein

**SSP**, standard spot number; **SD/LD**, normalized spot volume in SD divided by normalized spot volume in LD, from 6 different plants; **Pval**, *p*-value; **Th *M*r**, theoretical molecular mass; **Exp *M*r**, experimental molecular mass; **Th p*I***, theoretical p*I*; **Exp p*I***, experimental p*I*; **Pep**, number of peptides mass and in ( ) the number of MS/MS ions matching the query; **M score**, MOWSE score; **% Cov**, percentage of coverage; **Function**, description of protein identity (*spots with multiple positive identifications)

**Table 2:** Proteins whose abundance was significantly more abundant in SD than LD at 7 days.

SSP	SD/LD	Pval	Th Mr	Exp Mr	Th Pi	Exp Pi	Pep	M score	% Cov	Function
SSP3313	5.39	0.01	39	43	5.4	5.3	19 (9)	768	70	Glutamine synthetase
SSP8826	10.7	0.04	115	95	6.9	6.2	34 (13)	797	37	Glycine dehydrogenase
SSP6605	16.6	0.00	64	75	5.7	5.8	29 (15)	651	38	Lysyl-tRNA synthetase
SSP8301	2.90	0.02	41	41	6.3	6.1	31 (17)	921	68	Alpha-1,4-glucan-protein synthase 1
SSP4719	22.8	0.00	83	69	6.8	5.4	33 (13)	707	36	Beta-D-xylosidase
SSP2209	3.15	0.03	29	36	5.3	5.1	15 (9)	511	48	L-galactose 1-phosphate phosphatase
SSP5502	3.18	0.04	52	58	5.1	5.5	31 (14)	965	69	ATP synthase beta chain 2, mitochondrial*
SSP4309	15.3	0.03	37	40	6.3	5.4	8 (6)	250	26	ATP synthase gamma chain 1t
SSP0008	5.62	0.00	28	25	5.1	4.8	9 (8)	537	44	Light-harvesting complex II LHCB1
SSP2101	4.25	0.02	27	26	5.2	5.0	10 (5)	236	36	Light-harvesting complex II LHCB1
SSP1008	4.55	0.00	28	25	5.7	4.9	7 (6)	290	19	Light-harvesting complex II LHCB2
SSP1308	3.45	0.02	52	46	5.5	4.9	26 (3)	304	44	RUBISCO activase, chloroplast
SSP4814	3.54	0.02	102	87	6.1	5.5	20 (9)	545	22	Lipoxygenase (LOX2)*
SSP2505	6.08	0.01	64	63	5.8	5.0	10 (5)	194	23	Chaperonin*
SSP0707	5.13	0.04	92	90	5.0	4.8	21 (8)	249	25	Endoplasmin precursor (GRP94)
SSP1701	5.59	0.05	92	89	5.0	4.9	32 (19)	990	37	Endoplasmin precursor (GRP94)
SSP1618	5.01	0.03	68	66	5.7	5.0	21 (11)	507	40	60 kDa chaperonin beta subunit
SSP1102	19.0	0.01	32	29	7.8	4.8	9 (7)	421	43	HrBP1-1 PAP/fibrillin family
SSP2404	4.95	0.01	50	53	4.9	5.0	31 (17)	1150	73	Tubulin alpha-3 chain
SSP0227	5.06	0.01	19	34	5.6	4.8	12 (8)	255	49	14-3-3 protein GF14 iota (GRF12)*
SSP0109	5.40	0.00	29	30	4.8	4.7	23 (12)	662	68	14-3-3 protein GF14 omega (GRF2)
SSP2708	2.67	0.01	90	87	5.1	5.1	39 (18)	1170	55	Cell division cycle protein 48
SSP6608	4.43	0.04	82	78	5.8	5.8	35 (14)	398	47	N-ethylmaleimide sensitive factor*
SSP1319	9.87	0.00	4	43	4.8	4.8	11 (4)	186	29	Eukaryotic initiation factor 3H1 subunit
SSP4815	9.07	0.00	99	85	6.1	5.5	52 (17)	1110	45	ATP-dependent Clp protease ATP-binding sub (CD4B)
SSP4603	3.80	0.04	71	76	5.2	5.4	29 (10)	518	40	Heat shock cognate 70 kDa protein 1
SSP2604	3.47	0.00	71	72	5.2	5.1	39 (16)	1080	47	Heat shock cognate 70 kDa protein 1
SSP1617	4.02	0.02	53	63	5.0	5.0	17 (4)	258	37	RNA recognition motif (RRM)-containing protein
SSP0018	65.8	0.01	19	17	4.5	4.5	8 (5)	247	32	Translationally-controlled tumor protein
SSP4008	10.1	0.00		21		5.5				

**SSP**, standard spot number; **SD/LD**, normalized spot volume in the SD divided by the normalized spot volume in the LD, from 6 different plants; **Pval**, *p*-value; **Th *M*r**, theoretical molecular mass; **Exp *M*r**, experimental molecular mass; **Th p*I***, theoretical p*I*; **Exp p*I***, experimental p*I*; **Pep**, number of peptides mass and in ( ) the number of MS/MS ions matching the query; **M score**, MOWSE score; **% Cov**, percentage of coverage; **Function**, description of protein identity (*spots with multiple positive identifications)

**Table 3:** Proteins whose abundance was significantly more abundant in LD than SD at 28 days.

SSP	SD/LD	Pval	Th Mr	Exp Mr	Th Pi	Exp Pi	Pep	M score	% Cov	Function
SSP7619	0.11	0.00	48	65	6.7	5.9	12 (3)	113	30	2-isopropylmalate synthase B
SSP7613	0.24	0.00	61	41	7.0	5.9	18 (6)	247	30	Dihydroxy-acid dehydratase*
SSP6614	0.19	0.04	63	63	6.4	5.7	14 (7)	366	21	Ketol-acid reductoisomerase*
SSP6517	0.35	0.00	63	61	6.4	5.8	17 (11)	770	33	Ketol-acid reductoisomerase
SSP6512	0.38	0.00	55	53	7.6	5.7	22 (8)	385	35	3-P-shikimate 1-carboxyvinyltransferase, chloroplast
SSP8723	0.41	0.00	85	79	6.1	6.3	38 (14)	908	52	5-Me-tetrahydropteroyltriglu-homocys S-Me-transferase
SSP8706	0.12	0.05	85	83	6.1	6.1	33 (12)	701	39	5-Me-tetrahydropteroyltriglu-homocys S-Me-transferase
SSP9702	0.38	0.01	85	83	6.1	6.4	25 (13)	783	39	5-Me-tetrahydropteroyltriglu-homocys S-Me-transferase
SSP5408	0.37	0.01	43	51	5.7	5.7	16 (8)	489	44	S-adenosylmethionine synthetase 1 (SAM1)
SSP6425	0.03	0.00	43	49	5.6	5.7	9 (7)	449	19	S-adenosylmethionine synthetase 1 (SAM1)
SSP5415	0.01	0.00	43	50	5.6	5.5	33 (9)	512	57	S-adenosylmethionine synthetase 1 (SAM1)
SSP5604	0.38	0.00	60	66	5.4	5.5	23 (14)	908	54	2,3-bis-P-glycerate-independent P-glycerate mutase
SSP5611	0.44	0.02	60	67	5.4	5.6	22 (12)	607	54	2,3-bis-P-glycerate-independent P-glycerate mutase
SSP7328	0.48	0.04	38	43	6.6	6.0	22 (9)	454	53	Glyceraldehyde-3-P dehydrogenase A, chloroplast*
SSP6405	0.11	0.01	42	45	6.3	5.7	13 (6)	346	42	Phosphoglycerate kinase, cytosolic*
SSP2307	0.33	0.02	36	41	5.0	5.1	11 (6)	175	32	Pyruvate dehydrogenase E1 sub beta*
SSP8414	0.25	0.02	46	51	6.0	6.3	28 (9)	480	54	Isocitrate dehydrogenase, chloroplast
SSP8316	0.36	0.01	35	40	6.2	6.1	21 (14)	865	65	Malate dehydrogenase, cytosolic
SSP5206	0.19	0.00	32	33	5.5	5.6	29 (3)	330	70	Phosphogluconate dehydrogenase
SSP4314	0.15	0.01	44	41	6.3	5.5	11 (6)	159	26	Transketolase
SSP8707	0.1	0.00	67	75	6.1	6.1	32 (16)	928	45	Transketolase, chloroplast
SSP8602	0.37	0.00	67	75	6.1	6.1	30 (12)	659	46	Transketolase, chloroplast
SSP7207	0.22	0.00	28	31	5.6	5.9	23 (4)	306	60	Phosphomannomutase
SSP3201	0.23	0.00	35	39	5.1	5.2	24 (7)	614	72	Fructokinase-1
SSP2205	0.46	0.03	35	39	5.1	5.1	20 (11)	656	61	Fructokinase-1*
SSP7401	0.09	0.00	43	47	5.9	5.8	20 (7)	349	51	GDP-mannose 3,5-epimerase 1
SSP7708	0.27	0.03	92	83	6.0	6.0	54 (16)	824	45	Sucrose synthase
SSP3502	0.17	0.00	59	56	6.6	5.2	21 (7)	355	45	ATP synthase beta chain 2, mitochondrial*
SSP4305	0.42	0.00	37	42	6.3	5.4	13 (3)	304	31	ATP synthase gamma chain 1t
SSP7114	0.14	0.02	25	26	5.8	5.9	8 (4)	203	32	Inorganic pyrophosphatase
SSP1519	0.42	0.01	54	60	5.0	5.0	18 (6)	247	43	V-type H+-transporting ATPase subunit B
SSP5708	0.45	0.00	92	84	5.5	5.6	35 (12)	844	46	Phospholipase D alpha 1*
SSP6423	0.05	0.02	54	49	7.0	5.8	18 (8)	343	32	Sulfate adenylyltransferase*
SSP7217	0.08	0.00	35	38	6.0	5.9	10 (6)	295	40	Thioredoxin reductase 2
SSP7313	0.25	0.00	37	41	5.8	5.9	12 (10)	517	28	Anthocyanidin reductase
SSP7325	0.11	0.00	26	42	7.6	5.9	7 (4)	156	35	Anthocyanidin reductase
SSP2120	0.28	0.00	25	27	5.3	5.2	9 (6)	428	59	Chalcone isomerase*
SSP6205	0.46	0.00	35	39	5.8	5.7	21 (14)	831	60	Cinnamyl alcohol dehydrogenase
SSP6413	0.11	0.00	40	48	5.6	5.8	17 (5)	447	46	Leucoanthocyanidin dioxgenase
SSP2406	0.3	0.02	50	53	5.0	5.1	23 (13)	705	53	Tubulin alpha chain
SSP2516	0.42	0.00	50	53	5.0	5.1	21 (13)	721	48	Tubulin alpha chain
SSP2404	0.15	0.01	50	53	4.9	5.0	31 (17)	1150	73	Tubulin alpha-3 chain
SSP1516	0.05	0.01	50	55	4.8	4.9	24 (5)	250	35	Tubulin beta-7 chain
SSP6104	0.25	0.00	27	25	8.8	5.7	8 (5)	281	44	Chaperonin 21, Chloroplast
SSP6609	0.27	0.01	57	65	5.6	5.7	24 (11)	361	54	Chaperonin containing TCP1, beta subunit
SSP5617	0.11	0.01	59	65	5.7	5.6	22 (5)	238	27	Chaperonin containing TCP1, epsilon subunit*
SSP8609	0.17	0.00	61	64	6.0	6.1	32 (9)	458	55	Chaperonin containing TCP1, eta subunit*
SSP6606	0.25	0.00	61	68	5.6	5.7	32 (13)	548	55	Chaperonin containing TCP1, gamma sb
SSP4517	0.31	0.00	59	64	5.5	5.5	20 (7)	275	46	Chaperonin containing TCP1, theta sb
SSP8605	0.48	0.00	59	63	6.0	6.1	42 (13)	566	59	Chaperonin containing TCP1, zeta subunit
SSP1702	0.15	0.00	92	89	5.0	4.9	26 (18)	517	30	Endoplasmin precursor GRP94
SSP3012	0.03	0.00	17	18	5.2	5.2	14 (7)	573	93	Peroxiredoxin-5
SSP8103	0.43	0.02	27	27	6.6	6.1	6 (3)	125	23	Proteasome 20 S alpha subunit C
SSP7108	0.11	0.00	27	26	6.1	6.0	26 (7)	407	61	Proteasome 20 S alpha subunit G
SSP2008	0.36	0.03	22	22	5.5	5.1	9 (5)	285	56	Proteasome 20 S beta subunit C1
SSP6014	0.35	0.00	22	22	5.9	5.8	21 (11)	623	74	Proteasome 20 S beta subunit D
SSP7520	0.09	0.01	47	53	5.9	5.9	10 (6)	141	25	Proteasome 26 S regulatory subunit RPN6
SSP0019	0.04	0.02	20	14	5.8	4.7	7 (3)	191	35	Ribosomal protein L12-1, chloroplast
SSP4212	0.17	0.00	37	37	5.6	5.5	3 (2)	82.5	12	Serine carboxypeptidase II
SSP2513	0.5	0.00	68	63	5.7	5.1	19 (12)	640	40	60 kDa chaperonin beta subunit
SSP2605	0.39	0.04	68	65	5.7	5.1	19 (10)	558	39	60 kDa chaperonin beta subunit
SSP0215	0.26	0.03	29	31	4.8	4.8	15 (7)	347	52	14-3-3 protein GF14 omega (GRF2)
SSP0216	0.12	0.01	29	30	4.8	4.7	23 (10)	554	51	14-3-3 protein GF14 omega (GRF2)
SSP0221	0.1	0.00	29	31	4.7	4.6	19 (8)	460	60	14-3-3 protein GF14 omega (GRF2)
SSP0211	0.13	0.00	29	31	4.7	4.7	24 (10)	562	59	14-3-3 protein GF14 omega (GRF2)
SSP3712	0.24	0.01	90	85	5.1	5.2	36 (17)	1080	47	Cell division cycle protein 48
SSP4514	0.35	0.00	50	54	5.4	5.5	22 (6)	324	42	RAB GDP dissociation inhibitor 1 ATGD1*
SSP1611	0.08	0.01	65	69	4.9	5.0	21 (7)	302	40	Ser/thr-prot phosphatase 2A 65 kDa reg sub A
SSP2701	0.29	0.00	65	81	5.0	5.0	28 (12)	793	42	heat shock protein 81-4
SSP6417	0.34	0.00	48	52	5.8	5.8	17 (8)	272	41	UVB-resistance protein UVR8*
SSP0204	0.31	0.02	28	35	4.5	4.5	12 (7)	426	45	elongation factor 1-beta
SSP1201	0.25	0.02	24	31	4.8	4.8	9 (5)	318	57	Elongation factor 1-beta 1
SSP7701	0.32	0.00	56	87	6.1	5.9	13 (7)	282	29	Elongation factor EF-2*
SSP4412	0.49	0.01	47	50	5.5	5.4	35 (15)	622	55	eukaryotic translation initiation factor 4A-1
SSP4408	0.38	0.00	47	52	5.5	5.4	32 (7)	215	50	eukaryotic translation initiation factor 4A-1
SSP4402	0.39	0.01	47	51	5.4	5.4	33 (9)	368	62	eukaryotic translation initiation factor 4A-2
SSP3603	0.32	0.03	71	72	5.2	5.2	29 (11)	464	42	Heat shock cognate 70 kDa
SSP1603	0.44	0.03	57	73	4.7	4.8	28 (14)	1240	52	Heat shock protein 70 kDa
SSP3122	0.06	0.01	27	28	5.1	5.3	13 (3)	199	65	Coatomer subunit epsilon
SSP6722	0.02	0.05	84	82	5.8	5.7	26 (9)	392	35	Protein transport protein Sec23A
SSP7204	0.19	0.00	33	31	5.8	5.9	14 (3)	229	42	Protein transport SEC13
SSP0101	0.3	0.00	31	27	5.4	4.6	7 (7)	206	23	RNA-binding protein cp29
SSP5221	0.11	0.02	33	37	5.6	5.6	7 (5)	135	31	Carboxyesterase 5 CXE5
SSP1707	0.06	0.02	90	84	4.9	4.8	41 (12)	822	46	Embryo defective 1956
SSP0304	0.45	0.00	35	42	4.7	4.6	11 (8)	485	39	Late embryogenesis abundant
SSP6116	0.16	0.04	27	29	5.5	5.7	11 (3)	219	41	Stem-specific protein TSJT1
SSP0017	0.03	0.04	19	17	4.5	4.6	4 (3)	117	20	Translationally-controlled tumor protein
SSP4317	0.08	0.00	40	43	5.8	5.4	20 (10)	419	47	Unknown protein*
SSP9604	0.21	0.00		50		6.4				
SSP8425	0.12	0.00		49		6.3				
SSP0120	0.07	0.00		27		4.7				
SSP0132	0.46	0.03		27		4.6				
SSP6711	0.23	0.03		88		5.7				

**SSP**, standard spot number; **SD/LD**, normalized spot volume in the SD divided by the normalized spot volume in the LD, from 6 different plants; **Pval**, *p*-value; **Th *M*r**, theoretical molecular mass; **Exp *M*r**, experimental molecular mass; **Th p*I***, theoretical p*I*; **Exp p*I***, experimental p*I*; **Pep**, number of peptides mass and in ( ) the number of MS/MS ions matching the query; **M score**, MOWSE score; **% Cov**, percentage of coverage; **Function**, description of protein identity (*spots with multiple positive identifications)

**Table 4:** Proteins whose abundance was significantly more abundant in SD than LD at 28 days.

SSP	SD/LD	Pval	Th Mr	Exp Mr	Th Pi	Exp Pi	Pep	M score	% Cov	Function
SSP6408	4.15	0.00	37	46	6.6	5.7	9 (6)	268	35	Fumarylacetoacetase*
SSP5407	17.8	0.00	48	46	7.1	5.7	7 (5)	193	15	Glutamine synthetase
SSP8713	2.69	0.01	115	92	6.9	6.2	35 (13)	723	36	Glycine dehydrogenase
SSP7412	3.21	0.00	48	49	5.9	6.0	25 (11)	523	48	Methylthioribose kinase
SSP6412	3.93	0.01	48	48	7.6	5.8	13 (6)	205	29	Glyceraldehyde-3-P dehydrogenase B, chloroplast*
SSP7317	3.4	0.00	43	41	8.1	6.0	18 (11)	781	45	Fructose-bisphosphate aldolase, chloroplast*
SSP5218	3.49	0.00	32	36	5.5	5.6	21 (9)	446	57	Phosphogluconate dehydrogenase
SSP7710	3.29	0.02	67	76	5.9	5.9	33 (10)	579	55	Transketolase, chloroplast
SSP5106	2.99	0.00	29	26	8.8	5.6	12 (4)	159	31	Dehydroascorbate reductase
SSP7406	4.28	0.00	47	47	6.3	5.9	24 (12)	829	59	Monodehydroascorbate reductase*
SSP4401	9.27	0.00	56	53	6.5	5.4	31 (10)	674	53	Glucose-1-P adenylyltransferase small sub, chloroplast
SSP5219	5.62	0.00	33	37	5.5	5.6	16 (7)	331	42	Lactoylglutathione lyase
SSP2202	2.96	0.00	40	33	6.2	5.0	10 (6)	203	24	phosphoglycolate phosphatase
SSP3111	6.23	0.00	35	30	8.7	5.2	6 (5)	136	21	phosphoglycolate phosphatase
SSP3501	8.93	0.00	55	62	5.3	5.2	22 (15)	752	39	ATP synthase CF1 alpha subunit
SSP2515	4.63	0.00	55	63	5.3	5.1	31 (16)	905	43	ATP synthase CF1 alpha subunit
SSP5314	5.84	0.00	33	41	5.7	5.6	20 (13)	677	79	Quinone oxidoreductase, chloroplast
SSP7321	8.32	0.00	33	40	5.7	5.9	10 (3)	173	49	Quinone oxidoreductase, chloroplast
SSP5009	3	0.01	21	16	6.9	5.6	3 (3)	220	19	Cyt B6-F complex iron-sulfur sub, PETC
SSP2206	2.82	0.01	35	31	6.1	5.1	11 (7)	268	27	Photosystem II PsbO protein
SSP1121	5	0.00	35	30	7.6	5.0	7 (3)	86	23	Photosystem II PsbO protein
SSP1116	3.02	0.02	35	31	7.6	5.0	8 (4)	100	28	Photosystem II PsbO protein
SSP3010	5.36	0.00	28	23	8.3	5.4	8 (6)	400	31	Photosystem II PsbP protein
SSP1006	12.1	0.00	26	22	5.8	5.0	5 (3)	125	18	Light-harvesting complex I LHCA1
SSP0008	9.59	0.00	28	25	5.1	4.9	9 (8)	537	44	Light-harvesting complex II LHCB1
SSP0006	4.45	0.01	29	24	5.1	4.8	5 (2)	107	12	Light-harvesting complex II LHCB3*
SSP1002	3.91	0.00	29	23	5.2	4.9	5 (2)	122	14	Light-harvesting complex II LHCB3
SSP1304	16.3	0.00	42	43	6.0	4.9	21 (14)	644	43	Sedoheptulose-1,7-bisphosphatase, Chloroplast
SSP8619	21.4	0.00	50	71	6.7	6.2	16 (4)	310	34	RUBISCO large subunit
SSP1306	3.24	0.01	48	46	5.5	4.9	27 (11)	588	50	RUBISCO activase, chloroplast
SSP1416	4.01	0.00	52	50	5.7	5.0	24 (11)	485	41	RUBISCO activase, chloroplast
SSP1411	6.01	0.00	52	50	5.5	4.9	27 (7)	546	47	RUBISCO activase, chloroplast
SSP1308	7.35	0.00	52	45	5.5	4.9	26 (3)	304	44	RUBISCO activase, chloroplast
SSP3304	9.41	0.00	52	45	5.5	5.3	22 (7)	281	43	RUBISCO activase, chloroplast
SSP1404	5.21	0.00	52	51	5.5	4.8	23 (8)	342	44	RUBISCO activase, chloroplast
SSP4407	4.74	0.01	91	51	8.6	5.4	11 (5)	177	11	Triacylglycerol lipase
SSP4206	5.18	0.00	34	35	5.4	5.4	13 (6)	440	49	Isoflavone reductase protein 4
SSP3002	5.67	0.00	25	24	5.5	5.2	4 (2)	46	26	Proteasome 20 S beta subunit A
SSP5606	12.3	0.00	75	70	5.5	5.6	9 (3)	216	19	Subtilisin protease C1
SSP5217	3.57	0.00	33	31	6.4	5.6	18 (6)	333	53	Thioredoxin-like protein CDSP32
SSP0205	4.65	0.00	35	35	5.1	4.6	9 (7)	290	26	PAP/fibrillin family
SSP1102	11.6	0.00	32	29	7.8	4.8	9 (7)	421	43	PAP/fibrillin family
SSP7009	4.98	0.02	17	16	6.0	6.0	10 (5)	272	49	pathogenesis protein 1
SSP3604	4.64	0.00	74	71	5.8	5.2	11 (6)	172	16	FtsH protease (VAR2)*
SSP2616	10.4	0.00	74	68	5.8	5.2	31 (11)	699	48	FtsH protease (VAR2)
SSP2609	4.1	0.00	74	70	5.8	5.1	17 (9)	405	21	FtsH protease (VAR2)
SSP0703	6.22	0.00	90	85	4.9	4.8	17 (5)	279	29	Embryo defective 1956
SSP8214	5.24	0.00	43	40	7.1	6.1	25 (8)	463	57	mRNA-binding protein precursor
SSP5011	5.73	0.00	28	19	5.6	5.6	6 (6)	216	23	Thylakoid lumenal 19 kDa, chloroplast
SSP0202	43	0.00		35		4.5				
SSP0402	2.88	0.00		47		4.6				
SSP3507	2.94	0.02		59		5.2				
SSP4511	3.09	0.00		62		5.5				
SSP3714	3.61	0.00		94		5.2				

**SSP**, standard spot number; **SD/LD**, normalized spot volume in the SD divided by the normalized spot volume in the LD, from 6 different plants; **Pval**, *p*-value **Th *M*r**, theoretical molecular mass; **Exp *M*r**, experimental molecular mass; **Th p*I***, theoretical p*I*; **Exp p*I***, experimental p*I*; **Pep**, number of peptides mass and in ( ) the number of MS/MS ions matching the query; **M score**, MOWSE score; **% Cov**, percentage of coverage; **Function**, description of protein identity (*Spots with multiple positive identifications)

**Figure 6:**
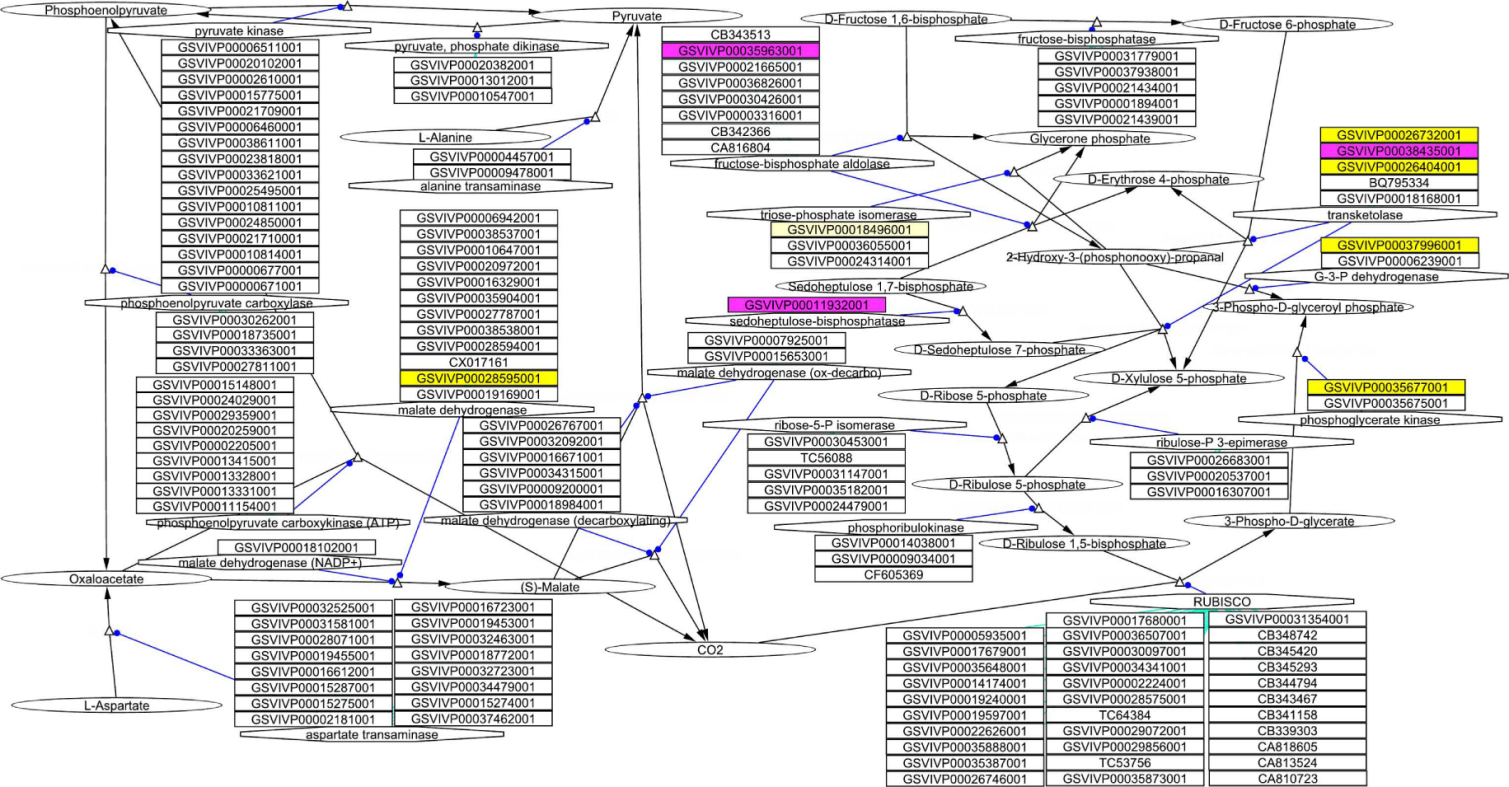
**Changes of *V. riparia *shoot tip protein abundance observed in the carbon fixation pathway in LD or SD treatment**. Protein IDs significantly abundant in different treatments are represented by color: proteins more abundant in 7LD (light yellow), proteins more abundant in 28LD (dark yellow) and proteins more abundant in 28SD (magenta). Other shapes in the pathway diagram indicate: function (hexagon), protein (rectangle), metabolite (ellipse), reaction node (open triangle), catalysis (blue lines with circle at the tip), and metabolic reaction (black lines with triangle at the tip).

Proteomic data was uploaded onto VitisNet as described by Grimplet et al. [18]. Supplementary material in Additional File 2 provides a subset of 15 VitisNet molecular networks, that can be opened in Cytoscape http://www.Cytoscape.org, showing proteins with outstanding expression between LD and SD. Fifteen networks revealed outstanding evolution of protein abundance in relation to photoperiod: 5 related to carbohydrate metabolism (vv10010 glycolysis, vv10053 ascorbate and aldarate metabolism, vv10500 starch and sucrose metabolism, vv10620 pyruvate metabolism, vv10770 pantothenate, and CoA biosynthesis), 3 linked to amino acid metabolism (vv10271 methionine metabolism, vv10290 valine, leucine, and isoleucine biosynthesis, vv10450 selenoamino acid metabolism), 4 correlated to energy metabolism (vv10190 oxidative phosphorylation, vv10195 photosynthesis, vv10196 photosynthesis antenna proteins, vv10710 carbon fixation (Figure 6)), 2 associated with secondary metabolism (vv10940 phenylpropanoid biosynthesis, vv10941 flavonoid biosynthesis), and 1 related to protein fate (vv23050 proteasome).

## Discussion

Decreasing daylength is the environmental signal utilized by many perennial plant systems to initiate growth cessation and to prepare for adverse environmental conditions associated with winter in temperate zones. In this study, *V. riparia *vines showed no difference in the rate of shoot growth in LD and SD during the first seven days of differential photoperiod treatments; thereafter, growth ceased in the SD treatment and the shoot apices senesced upon prolonged SD exposure. This data is in accordance with previous studies that found shoot length and node number were greater under long days [9,11,19-22].

Several proteins identified in this *V. riparia *study are in common with proteins identified in shoot or leaf proteome profiles of several *V. vinifera *and *V. rotundifolia *cultivars [23,24]. However, those studies indicated that genotype was the most significant factor determining differences in protein abundance [23]. Therefore, this study presents only the differentially abundant proteins in response to LD growth maintenance and SD induced growth cessation in *V. riparia*. In contrast to photoperiod studies in peach bark (*Prunus persica*) [25], which showed a small number (66) of proteins differentially abundant in response to SD, *V. riparia *had 216 proteins (≥ two-fold ratio, p-value ≤ 0.05) that showed differential abundance in response to SD. There were very few differentially abundant proteins in common between peach bark (a storage tissue) and grape shoot tip (predominately photosynthetic tissue) in response to photoperiod treatment. A comparison of the proteomes of the *V. riparia *shoot tissue exposed to LD and SD indicated a greater number of proteins in LD than in SD. Since an individual spot intensity is relative to the total intensity, this difference could be related to a higher abundance of a few major proteins in SD treatments, thus reducing the share of lower abundant proteins. In addition to differences in the number of abundant proteins, a comparison of the proteomes identified several molecular parameters that could play significant roles in plant adaptation to decreasing photoperiod.

### Carbon fixation and carbohydrate metabolism

Major changes were observed in the abundance of proteins involved in the carbon assimilation process and carbohydrate metabolism in relation to photoperiod treatment. Several enzymes involved in the Calvin-Benson cycle were more abundant in LD shoot tips (Table 1 and 3): phosphoglycerate kinase (SSP6405), chloroplastic glyceraldehyde-3-phosphate dehydrogenase A (SSP7328), triose phosphate isomerase (SSP9108), and four transketolase proteins (SSP7601; SSP4314; SSP8707; SSP8602). In contrast, Rubisco (SSP8619), seven Rubisco activases (SSP1308 (Table 1 and 4); SSP1306; SSP1416; SSP1411; SSP3304; SSP1404), fructose-1,6-bisphosphatase (SSP7317), another transketolase (SSP7710), and sedoheptulose-1,7-bisphosphatase (SSP1304) were more abundant in SD shoot tips (Table 2 and 4; Figure 6).

In barley shoot apices it was noted that the rate of carbohydrate production was considerably slower in 8 h than in 16 h photoperiods [26]. Similarly, in this study, enzymes involved in the reduction phases of the Calvin-Benson cycle are more abundant in LD shoot tips while enzymes involved in the carboxylation and regeneration phase are more abundant in SD shoot tips. In contrast, the greater recovery potential of ribulose-1, 5-bisphosphate exhibited in SD treatments may be related to an overall decrease in available carbon in comparison to the LD treatments.

Potentially higher carbohydrate availability in LD shoot tips may be responsible for the higher protein abundance of enzymes involved in glycolysis. These proteins include triose-phosphate isomerase (SSP9108), phosphoglycerate kinase (SSP6405), two phosphoglycerate mutases (SSP5604; SSP5611, both matching GSVIVP00033522001), pyruvate dehydrogenase (SSP2307), and pyruvate decarboxylase (SSP7608). In addition, the enzymes 2-isopropylmalate synthase (SSP7619) and malate dehydrogenase (SSP8316) (Table 1 and 3), which function in the pathway following glycolysis, were also more abundant in LD shoot tips.

Under LD conditions it appears that the carbon surplus promotes tissue growth by increasing the pyruvate pool. Roeske and Chollet [27] found that pyruvate accumulation was light dependent. The LD treated tissue had a greater abundance of sucrose synthase (SSP7708) enzymes. Similarly, an increase of sucrose synthase activity was observed in LD in soybean leaves [28], and a higher abundance of sucrose was observed in LD in tobacco leaves [29]. In *Arabidopsis*, enzymes in the glycolysis pathway showed a decrease in activity in conjunction with decreasing photoperiods, while activity of photosynthesis and starch synthesis remained high [30].

A greater abundance of enzymes leading to the accumulation of starch has been observed in SD shoot tips. Analysis identified these storage enzymes as fructose bisphosphate aldolase (SSP7317), a second glyceraldehyde-3-phosphate dehydrogenase (SSP6412), and glucose-1-phosphate adenylyltransferase (SSP4401). Previous reports illustrated that plants grown in shorter photoperiods or lower light intensities usually synthesize proportionally more starch [29,31,32]. The present study reveals a clear contrast in carbon utilization through its enzymatic steps. While more carbon is probably accumulated and used for the plant growth in LD, under SD plants appear to store the carbon as starch.

### Amino acid metabolism

Most minor amino acid abundance in plants has shown poor correlation with short term photoperiod changes [29,33]. These insignificant associations suggested that the variation in minor amino acids cannot be traced to short-term changes in primary carbon and nitrogen assimilation [33]. However, glutamate, glutamine, glycine, asparagine, alanine, threonine, and serine present daily variation in abundance in tobacco [29]. These authors also reported that all amino acids assayed were more abundant in LD than SD unless they could not be detected.

Glutamate acts at the center of nitrogen flow by incorporating ammonia into the plant [34]. Glutamine synthetases are especially important in the transport of nitrogen in aerial parts of the plant, and play different roles according to their cellular localization. Two glutamine synthetases have been detected as differentially abundant. One, presumably cytosolic (SSP4315), was more abundant in 7LD (Table 1), and the second, likely chloroplastic (SSP5407), was more abundant in 28SD. A third glutamine synthetase (GSVIVP00030210001) has been identified on two proximal spots (SSP3311; SSP3313); SSP3311 was more abundant in 7LD and SSP3313 was more abundant in 7SD. The differentiation between these glutamine synthetase spots is not likely caused by phosphorylation because SSP3313 has a slightly higher molecular weight (Mw) and the impact of phosphorylation on Mw is not generally noticeable in 2D gels. Over abundance of the cytosolic isoform in 7LD could be related to a greater nitrogen uptake in LD [35]. The major role of the chloroplastic isoform of glutamine synthetase in leaves is thought to be re-assimilation of the NH_3 _generated in photorespiration [36]. Glutamine synthetases are known to interact with 14-3-3 proteins [37]. Seven 14-3-3 proteins have been identified as differentially abundant in the present study, but only three correlate strictly with glutamine synthetase abundance. One 14-3-3 protein in LD (SSP0224) (Table 1) and two in SD (SSP0227; SSP0109) (Table 2) could also be involved in glutamine synthetase regulation during photoperiod.

In addition to chloroplastic glutamine synthetase, other enzymes involved in photorespiration [38] have been seen as more abundant in SD shoot tips. Decarboxylating glycine dehydrogenase (SSP8713) and two phosphoglycolate phosphatases (SSP2202; SSP3111) (Table 4) were differentially abundant in the 28 day treatments. Increased photorespiration in plants has been observed in the dark [39,40]. Photorespiration commonly produces reactive oxygen species (ROS), such as hydrogen peroxide (H_2_O_2_) [41], which can be toxic to plants at certain concentrations [42]. SD plants are known to cope better with H_2_O_2 _toxicity than LD plants [43]. Overabundance of enzymes in SD tissue related to ascorbate metabolism, which is involved in the detoxification of reactive oxygen species [41], also supports the hypothesis that the grapevine leaves have a higher level of peroxides under SD treatments. Monodehydroascorbate reductase (NADH) (SSP7406), dehydroascorbate reductase (SSP5106), and L-galactose 1-phosphate phosphatase (SSP2209) (Table 4), enzymes related to ascorbate biosynthesis, were all found in greater abundance in SD shoot tips. ROS such as H_2_O_2 _often elicit various physiological processes as signal molecules. H_2_O_2 _is produced during photosynthesis and photorespiration, and interacts with thiol-containing proteins. H_2_O_2 _directly activates numerous signaling pathways and transcription factors that regulate gene expression. Most research discusses the role of hydrogen peroxide in photorespiration and stress signaling, but it was not until recently that H_2_O_2 _was linked with cell growth and other cellular processes [41,44]. Hydroxyl radicals may have an active role in cell wall loosening [45]. Fry and colleagues suggest that ascorbate, H_2_O_2_, and copper ions (Cu^+2^) could interact to form OH radicals that actively loosen cell walls [46-48].

Additional enzymes involved in the metabolism of amino acids have been identified as more abundant in LD shoot tips (Table 1 and 3), possibly linked to a greater requirement of metabolites during growth. Aspartate semialdehyde dehydrogenase (SSP4320) forms an early branch point in the metabolic pathway forming lysine, methionine, leucine, and isoleucine from aspartate [49]. Enzymes involved downstream in the amino acids biosynthetic pathways have also been identified, including two ketol-acid reductoisomerase spots (SSP6614 and SSP6517, both matching GSVIVP00018719001) and dihydroxy-acid dehydratase (SSP7613), which are involved in the biosynthesis of isoleucine and valine. Furthermore, the 5-me-tetrahydropteroyltriglu-homocys S-Me-transferase (SSP8731;, SSP8726; SSP9702; SSP8718; SSP8723; SSP8706, all matching GSVIVP00003836001) and S-adenosylmethionine synthetase (SSP5408 matching GSVIVP00019707001; SSP6425; SSP5415 matching GSVIVP00028192001) are involved in methionine metabolism. Additionally, a cysteine synthase (SSP6307) has also been identified as more abundant in LD shoot tips.

### Secondary metabolism

Phenylpropanoid biosynthetic pathways provide anthocyanins for pigmentation, which are important compounds for protection against UV photo-damage in plants [50]. Effects of light treatment on phenylpropanoids have been widely studied in grape berries because of their important organoleptic properties. UV is known to increase phenolic composition in grape berries [51], and photoperiod has been identified as directly affecting the flavonoid composition. Flavonoid compounds decreased in SD versus LD in *Xanthium*, including anthocyanidin (quercitin), caffeoyl quinic acid, and bulk phenols [52]. In this study, three enzymes involved in the flavonoid biosynthesis were more abundant in LD shoot tips (Table 1 and 3): chalcone synthase (SSP8417), chalcone isomerase (SSP2120), and leucoanthocyanidin dioxgenase (SSP6413). Polyphenols, which also play an important role in protection against oxidation, and anthocyanidin reductase (SSP7313; SSP7325) were more abundant in LD shoot tips. Cinnamyl alcohol dehydrogenase (SSP6205), an enzyme that catalyzes the final step for production of lignin monomers, was also more abundant in LD shoot tips. Both cinnamyl alcohol dehydrogenase and lignin content have been shown to be enhanced by light in *Pinus radiata *callus cultures [53] and *Arabidopsis *roots [54].

### Energy metabolism

Surprisingly, a large number of proteins involved in photosystem II (PSII) (SSP2206; SSP1121; SSP1116; SSP3010), light harvest complex (LHC) subunit (SSP2101; SSP1008; SSP0008; SSP0006; SSP1002), and one involved in photosystem I (SSP1006) were more abundant in SD shoot tips (Table 2 and 4). These observations were unexpected since photoassimilate incorporation related proteins are more abundant in LD shoot tips (see previous carbon fixation section). However, several explanations are possible for these observations. Light stress-related oxidative damage causes protein degradation in PSII [55] and it could potentially be more dramatic under LD, leading to fewer PSII proteins. It is also noted that the leaves in the SD shoot tip are older than those in LD, since shoot growth ceases in the SD treatment and the LD shoot continues to grow and initiate new leaves. Thus the SD leaves may simply contain a greater number of photosystem complexes. Fewer photosystems does not necessarily reflect a decreased efficiency of the photosynthetic system, but rather an indication of leaf maturity and the fact that the photoassimilates are exported from the older mature leaves to the shoot tips. Mor and Halevy [56] and Lepistö et al. [57] observed a similar pattern in LHC proteins in rose (*Rosa*) shoots and *Arabidopsis *leaves respectively and showed that the photochemical efficiency of PSII was not affected by day length.

### Protein fate

The chaperonin TCP-1 is involved in cytoskeleton organization and keeps cytoskeletal proteins folded. Six of the eight subunits of the chaperonin TCP-1 complex were more abundant in 28LD shoot tips (SSP6609; SSP5617; SSP8609; SSP6606; SSP4517; SSP8605), (Table 3). Actin and tubulin monomers both interact with TCP-1 in order to reach their native states. Brackley and Grantham [58] and Himmelspach et al. [59] observed that abundance of TCP-1 subunits is age dependent but not growth dependent. This suggests that the greater abundance of TCP-1 subunits in the LD shoot tips was more related to the fact that the tissues are younger in the actively growing LD shoot tips than in the SD shoot tips. Consistently, tubulin proteins (SSP2406; SSP2516; SSP2404; SSP1516) (Table 1 and 3) were more abundant in LD shoot tips. Also, seven proteasome subunits were more abundant in LD (SSP2221; SSP4520; SSP8103; SSP7108; SSP2008; SSP6014; SSP7520). Proteasome plays an important role in plant life cycle processes; among them, cell division, growth and light signaling, which would all be higher in the actively growing LD shoot tip [60].

## Conclusions

Previous woody plant studies on photoperiod influence of protein abundance have focused predominately on specific bark storage and dehydrin proteins [61,62]. Studies of photoperiod induced changes in proteins during the induction of poplar bud dormancy showed many changes in protein profiles; however they did not identify the metabolic pathways involved in response to SD [63]. The proteome of *V. riparia *shoot tip tissue changes dramatically upon exposure to shorter photoperiod, although effects were more visible at 28 days than at 7 days. *V. riparia g*rapevines seem to shift the direction of carbon flux from metabolites for shoot growth in LD to starch accumulation when shoot growth ceases in SD. Both cytoskeletal proteins and protein fate enzymes were more abundant in LD shoot tips, suggesting turnover and production related to cell development. In addition, under LD there was a greater abundance of phenylpropanoids which may contribute to increased cell wall synthesis as a result of active growth. In contrast, photosystem proteins were more abundant in SD shoot tips which may be a factor of difference in leaf age as growth ceases in the SD treatment while the LD shoots continue to grow and produce new leaves. Abundance of photorespiratory enzymes was higher in SD shoot tips suggesting that reactive oxygen species were more abundant. This suggestion is also supported by an abundance of ascorbate metabolite enzymes which are involved processes for detoxifying reactive oxygen species.

## Methods

### Plant material and growth conditions

Potted, spur-pruned two to six-year-old *V. riparia *grapevines were removed from cold storage on 3/26/2007 and 3/24/2008. The plants were repotted and grown under a long photoperiod (LD, 15 h) with 25/20°C + 3°C day/night temperatures and 600 to 1400 μmol^m-2s-1 ^photosynthetic photon flux (PPF) in a climate-controlled, un-shaded glass greenhouse (En Tech Control Systems Inc., Montrose, MN) in Brookings, SD, USA (44.3°N). After 30 days, when grapevines reached 12-15 nodes, pots were randomized into two replicated treatment groups. Five days after randomization, differential photoperiod treatments began with one treatment group continuing in LD and the other receiving a short photoperiod (SD, 13 h). The SD was imposed using an automated, white covered black-out system (Van Rijn Enterprises LTD, Grassie, Ontario, Canada). At 7 and 28 days of differential photoperiod treatment, four-node shoot tips were collected between 8:30 and 10:30 AM, immediately frozen in liquid nitrogen, and placed at -80°C for future protein extraction. Three replications (5 vines/replication) were harvested in two consecutive years (2007 and 2008) resulting in a total of six replications analyzed by 2-D gel electrophoresis.

### Growth measurement

Shoot growth was measured weekly in both photoperiod treatments. Primary shoot length (in centimeters) and node number were recorded on a random sample of 11 LD and 11 SD *V. riparia *grapevines.

### Protein extraction

Protein extractions were performed in sets of four random samples. To reduce the effect of systematic variation in the extraction, only one randomly selected replicate of each condition was extracted at a time. These precautions reduced the occurrence of false positives but may have increased the variability between replicates. One gram of shoot tissue was ground to a fine powder in liquid nitrogen with a mortar and pestle. Extraction was adapted from the phenol-extraction protocol as described by Vincent et al. [14]. Ten mL of Hurkman extraction buffer [64] was added to each sample (0.7 M sucrose, 0.5 M Tris-HCl pH = 7.5, 50 mM EDTA, 0.1 mM potassium chloride, 2% 2-mercaptoethanol, 2 mM PMSF, 1 antiprotease tablet (Roche Diagnostics, Indianapolis, IN, USA)), homogenized for 30 sec, and incubated for 10 min at 4°C. After incubation an equal volume of 1 M Tris-saturated phenol (pH = 7.5) was added to each sample. The mixture was homogenized and incubated at 4°C for 30 min. The phases were separated by centrifugation (30 min, -4°C, 3,650 × *g*). The upper phenol phase was collected and re-extracted with an equal volume of Hurkman extraction buffer. The sample was vortexed, incubated at 4°C for 30 min, and centrifuged (30 min, -4°C, 3,650 × *g*). The upper phenol phase was collected, and five volumes of 0.1 M ammonium acetate in cold methanol (MeOH) were added to precipitate proteins. The samples were incubated overnight at -20°C and then centrifuged (30 min, -4°C, 3,650 × *g*). The pellet was washed twice with 5 mL of cold 0.1 M ammonium acetate/MeOH, twice with 10 mL of cold acetone, and once with 1.5 mL of cold acetone. The pellet was then vacuum-dried for 2 min and resolubilized in 1.5 ml of Rehydration Buffer (7 M urea, 2 M thiourea, 4% CHAPS, 20 mM DTT, 1% IPG buffer pH 4-7). Each sample was vortexed, allowed to stand at 4°C for 2 h to resolubilize proteins, and subsequently stored at -80°C.

### Protein assays

Protein concentrations were determined using an EZQ™ Protein Quantitation Kit (Invitrogen, Carlsbad, CA, USA), with ovalbumin as a standard according to manufacturer's instructions. Protein concentrations ranged from 1.6 to 5.9 mg/ml.

### 2D and gel staining

The 2D SDS-PAGE protocol was adapted from O'Farrell [65]. Iso-electric focusing (IEF) was carried out using immobilized pH gradient (IPG) strips (24 cm, pH 4-7, Immobiline™ DryStrip, GE Healthcare, Piscataway, NJ, USA). Samples were thawed on ice and centrifuged (15 min, 4°C, 10,000 × *g*) prior to loading on IPG strips. A loading volume of 450 μL of protein extract, corresponding to a protein amount of 1.0 mg, was added to each strip. Three mL of mineral oil was added to each well before IEF. Protein IEF was performed at 20°C using a Protean^® ^IEF Cell (Bio-Rad, Hercules, CA, USA) as follows: active rehydration at 50 volts (V) for 12 h, 200 V for 30 min with a linear increase in voltage, 500 V for 30 min with a linear increase in voltage, 1000 V for 1 h with a linear increase in voltage, and 10,000 V with a rapid increase in voltage until a total of 95,000 Volt-hours (Vh) was reached. Strips were then stored at -20°C until further use. Once thawed, the strips were washed for 20 min in an Equilibration Buffer (6 M urea, 30% v/v glycerol, 2 M Tris-HCl pH 8.8, 2% w/v SDS) containing 1% w/v DTT, followed by washing for 20 min with an Equilibration Buffer containing 2.5% w/v iodoacetamide. SDS-PAGE was performed using non-commercial 12% polyacrylamide gels (25 cm × 20 cm × 1 mm) and run at 40 V for 2 h and 120 V for 13 h in a Bio-Rad Protean^® ^II XL 2D Multi-Cell. A coomassie brilliant blue (CBB) G-250 procedure was used to stain the 2D gels. The gels were washed twice in 50% ethanol (EtOH)/2% phosphoric acid/de-ionized water (diH_2_O) v/v/v for 1 h, transferred to 2% phosphoric acid for 60 min, then washed in 17% ethanol/2% phosphoric acid/15% ammonium sulfate v/v/w for 1 h, and finally agitated for 3 days in 17% EtOH/15% ammonium sulfate/2% phosphoric acid/0.02% CBB G-250/diH2O v/w/v/w/v. The 2D gels were imaged using a ScanMaker 9800XL with TMA scanner (Microtek, Hsinchu, Taiwan).

### Protein analysis and statistical analysis

Gels from 7LD, 7SD, 28LD and 28SD treatments were compared using PDQuest™ Gel Analysis SW (Bio-Rad) with six replicates (three from each year of harvested plant material). Spots were matched within gels. If no spot was detected, a background value was used for the corresponding area in order to limit the rate of false positives. Average CV was calculated for each experiment. Differences in spot abundance were statistically evaluated using the ANOVA method with GeneANOVA software [66]. The number of detected spots showing differences with a p-value of ≤0.05 was determined. The spots were conserved only if their normalized intensity was higher than 0.01% of the total spot intensity. Differentially abundant spots were manually curated with respect to spot quality (e.g., sharpness, resolution) and the quality of spot matching to reduce false positives. Only spots with ≥ two-fold ratio between photoperiod conditions were conserved.

### Protein identification

Spot excision was performed manually, and then trypsin digested according to Rosenfeld et al. [67] using the Investigator™ ProPrep™ (Genomic Solutions, Ann Arbor, MI, USA). The tryptic fragments were analyzed using an ABI 4700 Proteomics Analyzer (Applied Biosystems, Foster City, CA, USA) MALDI TOF/TOF™ mass spectrometer (MS). A 0.5 mL aliquot of a matrix solution containing 10 mg/mL alpha-cyano-4-hydroxycinnamic acid (Sigma-Aldrich, Inc., St. Louis, MO, USA) and 10 mM ammonium phosphate (Sigma-Aldrich) in 70% acetonitrile was co-spotted with 0.5 mL of sample. Data were acquired in reflector mode from a mass range of 700 to 4,000 Da, and 2,500 laser shots were averaged for each mass spectrum. Each sample was internally calibrated if both the 842.51 and 2211.10 ions from trypsin autolysis were present. When both ions were not found the instrument used the default calibration. The twenty most intense ions from the MS analysis, not present on the exclusion list, were subjected to MS/MS analysis. The mass range was 70 to precursor ion with a precursor window of 21-3 Da and an average of 5,000 laser shots for each spectrum. The resulting file was then searched by using automated MASCOT software http://www.matrixscience.com/ through the IDQuest (Bio-Rad) interface to search the putative proteins obtained from the grapevine PN40024 homozygote genome [68], the Pinot Noir heterozygote genome [69], and the tentative contigs from the DFCI gene index ver. 5.0 http://compbio.dfci.harvard.edu/tgi/; ver. 18_9_2006, 23,871 sequences). Peptide tolerance was 20 ppm; 1 missed cleavage was allowed; MS/MS tolerance was 0.8 Da.

## Competing interests

The authors declare that they have no competing interests.

## Authors' contributions

KJV carried out the experiments and drafted the manuscript. AYF and JG participated in the design of the study and drafted the manuscript. All authors have read and approved the final manuscript.

## Supplementary Material

Additional file 1 **additional data for differentially expressed spots**. **SSP**, standard spot number; **SD/LD**, normalized spot volume in the SD divided by the normalized spot volume in the LD, from 6 different plants; **Pval**, *p*-value; **Average SD**, average intensity value in SD; **Average LD**, average intensity value in LD; **Exp *M*r**, experimental molecular mass; **Exp p*I***, experimental p*I*; **Th *M*r**, theoretical molecular mass; **Th p*I***, theoretical p*I*; **Pep**, number of peptides mass and in ( ) the number of MS/MS ions matching the query; **M score**, MOWSE score; **% Cov**, percentage of coverage; **Function**, description of protein identity. **8×**, protein ID in the gravevine genome with a 8× coverage; **12×**, protein ID in the grapevine genome with a 12× coverage; **Gels nomenclature**: first character, 7 or 28 for the date; second character, S or L for SD or LD; third character, 1, 2, 3 for the replicate number; fourth character, 7 or 8 for the harvested year (2007 or 2008) Click here for file

Additional file 2 **Cytoscape session containing the VitisNet molecular networks with proteins presenting outstanding evolution between LD and SD**. The session contains a subset of 15 VitisNet molecular networksshowing differential LD and SD protein expression. Tutorial for using VitisNet in Cytoscape can be obtained at http://vitis-dormancy.sdstate.org. Click here for file
